# A Type of Low-Latency Data Gathering Method with Multi-Sink for Sensor Networks

**DOI:** 10.3390/s16060923

**Published:** 2016-06-21

**Authors:** Chao Sha, Jian-mei Qiu, Shu-yan Li, Meng-ye Qiang, Ru-chuan Wang

**Affiliations:** 1 College of Computer, Nanjing University of Posts and Telecommunications, Nanjing 210003, China; qiujian_m@163.com (J.Q.); lisy_njupt@163.com (S.L.); qiangmy_njupt@163.com (M.Q.); wangrc@njupt.edu.cn (R.W.); 2 Provincial Key Laboratory for Computer Information Processing Technology, Soochow University, Suzhou 215006, China; 3 Jiangsu High Technology Research Key Laboratory for Wireless Sensor Networks, Nanjing 210003, China; 4 Nanjing University of Information Science & Technology, Nanjing 210044, China; 5 School of Engineering and Computation, New York Institute of Technology, New York 11568-8000, NY, USA

**Keywords:** Sensor Networks, mobile Sinks, low-latency, balance of energy consumption, redundancy on network coverage

## Abstract

To balance energy consumption and reduce latency on data transmission in Wireless Sensor Networks (WSNs), a type of low-latency data gathering method with multi-Sink (LDGM for short) is proposed in this paper. The network is divided into several virtual regions consisting of three or less data gathering units and the leader of each region is selected according to its residual energy as well as distance to all of the other nodes. Only the leaders in each region need to communicate with the mobile Sinks which have effectively reduced energy consumption and the end-to-end delay. Moreover, with the help of the sleep scheduling and the sensing radius adjustment strategies, redundancy in network coverage could also be effectively reduced. Simulation results show that LDGM is energy efficient in comparison with MST as well as MWST and its time efficiency on data collection is higher than one Sink based data gathering methods.

## 1. Introduction

For densely deployed Wireless Sensor Networks (WSNs), a typical way to send data is to the static Sink by multi-hop or points-to-point transmission [1,2,3,4]. However, nodes near the Sink tend to consume more energy as they are responsible for receiving and forwarding data from the whole network [5]. This easily leads to network disconnection and causes “hotspot problem” [6,7,8,9,10]. On the other hand, to reduce the end-to-end delay in data collection, Sink should be near the source of the event [11]. As the events occur at different regions within the network area, such an optimization cannot be achieved by using a static Sink [12].

Recently, how to use one or more mobile elements for data collection has become an important research topic [13]. With the help of one or more mobile Sinks, performance of WSNs in terms of energy consumption and coverage could be greatly improved. Francesco *et al.* [1] proposed that, due to the limited power of nodes, using one or more mobile Sinks for data collection in WSNs is an effective way to prolong network lifetime. Moreover, owing to the short communication distance between nodes and the mobile Sink, packet loss rate also decreases [14,15]. Besides, mobile Sinks may collect data from isolated regions which could improve the connectivity of the network.

A real-time data collection method is proposed in [16]. Trajectories of the mobile Sinks are designed by modeling the problem as the Traveling Salesman Problem (TSP) to achieve a balance between energy consumption and data collection latency. While another type of data collection method with the help of multiple mobile Sinks is proposed by Ren *et al.* [17] which reduces not only the energy consumption of the whole network but also transmission delays. To further reduce energy consumption of data collection, Gao *et al.* [13] divided the sensor nodes into sub-Sink nodes which are in a direct communication area (DCA) or far-away nodes that are within the distance of the multi-hop communication area (MCA). Sink moves along the fixed path to gather as much data as possible. In [18], path constrained Sink mobility is used to improve the energy efficiency of the single-hop sensor networks which may be infeasible due to the limits of the path location and communication power.

Nevertheless, how to find out the optimal moving paths for multiple Sinks and how to further reduce energy consumption and time delay on data uploading are still key problems that need to be discussed further.

The remainder of this paper is organized as follows: the related works, as well as the network model are described in Section 2 and Section 3 respectively. In Section 4, we analyze the sleep scheduling and sensing radius adjustment strategies in detail. Trajectories of the Sinks and the transmission path selection algorithm are also described in this section. Experimental results are shown in Section 5 and the conclusion is provided in the last section.

## 2. Related Works

With the development of sensor networks, how to improve the efficiency of data collection has become one of the hot spots of research. Heinzelman *et al.* [19] described the Low-energy Adaptive Clustering Hierarchy (LEACH) protocol for data collection in WSNs. Each node randomly decides whether or not to become a Cluster Head (CH) and the parameter used in decision making is the percentage of desired CHs. The CHs assign to each sensor from their cluster, a time slot for reporting, aggregate data received from individual sensors and send them directly to the Sink. While the major problem with LEACH is that the Sink may be very far from many CHs, therefore direct reporting may be extremely energy-consuming, or even impossible. 

Another type of cluster based data gathering algorithm named Stable Election Protocol (SEP) has been proposed in [20,21]. The network is divided into several clusters to collect data respectively and only the cluster headers need to communicate with the static Sink. Simulation results have shown that SEP is suitable for WSNs with a non-uniform distribution but nodes near the Sink or the cluster header die quickly, which caused an unbalance of energy consumption.

A type of Equalized Cluster Head Election Routing Protocol (ECHERP) for data collection in sensor networks is proposed by Nikolidakis, S.A. *et al.* [22]. It also pursues energy conservation through balanced clustering. ECHERP models the network as a linear system and using the Gaussian elimination algorithm, calculates the combinations of nodes that can be chosen as cluster heads in order to extend the network lifetime. Kandris, D. *et al.* focused on hierarchical routing in WSNs by using a new enhanced reactive protocol that aims at the achievement of power conservation through energy efficient routing [23]. This protocol performs the cluster leadership in a non-randomized way but takes into account the residual energy of nodes. Moreover, the data routing is based on a route selection policy which takes into consideration both the energy reserves of the nodes and the communication cost associated with the potential paths. 

In addition to the cluster based protocols, data uploading algorithms based on the tree-structure have also been widely used in WSNs. A type of data gathering method with the help of a Minimum Spanning Tree (MST) is proposed in [24]. Compared with other multi-hop data transmission methods, time delay in MST is lower and the efficiency on data aggregation is also enhanced. However, there is only a static Sink located at the edge of the network which could lead to unbalanced energy consumption. Sia, Y.K. *et al.* [25] presented a Spanning Multi-Tree Algorithm for Load Balancing (SMT-LB). SMT-LB aims to balance the load in multi-sink wireless sensor networks with heterogeneous traffic generating nodes. Simulation results show that SMT-LB is able to achieve a longer network lifetime with lower end-to-end delay by deploying sink nodes at the center of both left and right edges. In [26], the problem of building cluster-based topology for Sensor Networks with several sinks is also considered. The topology associated with each sink is modeled as an Independent Dominating Set with Connecting requirements (IDSC). Thus, the solution is a partition of a given graph into as many IDSC as there are sinks. In addition, several optimization criteria are proposed to implicitly or explicitly balance the topology. Network lifetime is improved since it benefits from a clustered structure and the number of hops control.

Furthermore, it becomes necessary to choose an appropriate aggregation factor and transmission radius depending upon the requirement of the application for which the WSN has been installed. Ghosh, K. *et al.* [27] presented a Fermat point based data aggregating protocol which is distance vector protocol in nature. Moreover, Gavalas, D. *et al.* introduces a novel algorithmic approach for energy-efficient itinerary planning of Mobile Agents (MA) engaged in data aggregation tasks [28]. It adopts an iterated local search approach in deriving the hop sequence of multiple traveling MAs over the deployed source nodes.

In recent years, the use of a mobile Sink which moves on a pre-specified trajectory has been extensively investigated by several researchers [13,14,29]. In the prospective of the mobility of mobile Sink, the works are divided into three categories [30,31]: Random Mobility; the movement of mobile Sink is on a random trajectory; Fixed Mobility, where the Sink moves on a fixed pre-specified path; Controlled Mobility, where the trajectory of the mobile Sink is controlled, depending on its position and density of data in its vicinity.

### 2.1. Random Mobility 

For the random mobility scheme, the mobile Sinks are often mounted on people or animals moving randomly to collect the required information [13]. However, due to its random mobility, it is difficult to bind the data transfer latency with the data delivery ratio [13]. Data is stored at the node temporarily to be transmitted when the mobile Sink is at the most favorable location in order to achieve the maximum network lifetime.

Jain *et al.* [32] proposed a type of data gathering method with the help of one mobile Sink. Due to the randomness of trajectory, the Sink could communicate with each node with the same probability. However, this method may increase the load of hot spots and the random movement of Sink will cause frequent breakage as well as reconstruction of communication link for the network. Moreover, it will also increase extra energy consumption and time delay.

A Virtual Circle Combined Straight Routing (VCCSR) protocol for data collection with a mobile Sink is proposed in [33]. VCCSR selects a set of cluster heads located near the virtual backbone. When the Sink issues a query in the network, a spanning tree is constructed to collect and complete data periodically. The goal of the proposed algorithm is to decrease the reconstruction cost and increase the data delivery ratio. However, a large number of nodes participate in routing, increasing network overhead.

In addition, Yu *et al*. proposed an elastic routing algorithm to minimize the traffic and enhance the reliability of data uploading from nodes to the mobile Sink [34]. When a Sink moves, the new location information is propagated along the reverse geographic routing path to the source during data delivery. However, this hop-by-hop data transmission could also increase the latency in data collection.

### 2.2. Fixed Mobility

A rendezvous-based data collection approach is proposed in [35] to select the optimal fixed path due to the delay limitation in WSNs with a mobile Sink. The mobile Sink visits exact locations called rendezvous points, according to the pre-computed schedule to collect data. The rendezvous points buffer and aggregate data originated from the source nodes through multi-hop relay and transfer to Sink on arrival. However, this method is only suitable for uniform distributed networks and the multi-hop relay mode will easily cause imbalances in energy consumption.

Somasundara *et al.* [36] proposes another type of mobile sensor networks with a path-fixed Sink supporting multi-hop communication. A speed control algorithm of the mobile Sink is suggested to improve the energy performance and a Shortest Path Tree (SPT) is used to choose the cluster heads and route data, which may cause imbalance in traffic and energy dissipation.

Seung-Wan and In-Seon proposed a Minimum Wiener index Spanning Tree (MWST for) as the routing topology network. Sensing data is collected by multiple mobile nodes [37] and the shortest path could also be found that effectively reduces energy consumption. Nevertheless, due to the multi-hop data transmission, nodes near the mobile Sinks run out of energy quickly.

### 2.3. Controlled Mobility

As for a controlled mobility scheme, it is possible to guarantee the data delivery efficiency with the help of efficient communication protocols and data collection schemes while the trajectories of the mobile Sinks are constrained or controllable [13]. The primary objective to control the mobility is to improve data gathering performance of the mobile Sink, yet handling the mobility of the Sink in a controlled manner is much more challenging than that of a random scheme [38]. Thus, most of the current work in this case has focused on how to design the optimal trajectories of mobile Sinks to improve the network performance [13].

In [39], a routing protocol, called MobiRoute, is suggested for WSNs with a path predictable mobile Sink to prolong the network lifetime and improve the packet delivery ratio. The Sink sojourns at some anchor points and the pause time is much longer than the movement time. Moreover, in MobiRoute all nodes need to know the topological changes caused by the Sink mobility which is unrealistic [13].

Somasundara *et al.* [40] have studied the scheduling problem of mobile Sink in WSNs to optimize the path of the Sink to visit each node and collect data before buffer overflows occur. Furthermore, a partitioning-based algorithm is presented in [40] to schedule the movements of the mobile element to avoid buffer overflow. However, the mobile Sinks in [40] need to visit all nodes to collect data which causes huge energy consumption.

A type of Mobile Sink based algorithm for Stable Election (MSE) is proposed by Wang *et al.* [16]. Sink moves back and forth along a certain designed trajectory which solves the energy hole problem. Moreover, MSE has also been proved to be used in a non-uniform network. However, nodes near the static trajectory die more quickly than others, especially in large networks. 

Kinalis *et al.* [41] propose a biased, adaptive Sink mobility scheme that adjusts to local network conditions, such as the surrounding density, remaining energy and the number of past visits in each network region. Sink moves probabilistically, favoring less visited areas in order to cover the network area faster, while adaptively staying longer in network regions that tend to produce more data. Nevertheless, the Sink usually takes a long time to finish a complete data gathering task that causes high latency problem.

The contributions of this paper can be concluded as follows: Firstly, with the help of the minimum network coverage model in WSNs, the network is divided into virtue regions and the leaders are selected according to their residual energy as well as the average distance to all of the other nodes. Network lifetime could be prolonged by periodically selection of the leader and the number of traverse points in LDGM is less than in other mobile Sink strategies. Secondly, the sleeping scheduling and the sensing radius adjustment strategies are adopted in LDGM that not only reduce the number of active nodes in data collection but also decreases the energy consumption on sensing. Moreover, with the help of multiple mobile Sinks, the end-to-end delay in data uploading is greatly reduced.

## 3. Network Model

As mentioned above, in sensor networks, random mobility and the fixed mobility schemes for data gathering are unable to balance energy consumption of nodes and the path for data gathering is not the optimal. While, the controlled mobility scheme in data gathering performs well in prolonging network lifetime as well as reducing time delay and could efficiently mitigate the energy hole problem. Thus, it is adopted as the mobile strategy in LDGM.

It is assumed that the network is a rectangular area whose length and width is *M* and *L* respectively. Nodes are deployed randomly in the area and meet the following conditions:

(1) Nodes are in static and high-density deployment. Total number and density of them is defined as *n* and *ρ* respectively.
(2) All nodes except the Sink have the same initial energy *E_0_*. The transmission power as well as the sensing radius of each node is also adjustable.
(3) There are several mobile Sinks in the network whose energy and storage space are unlimited.

**Figure 1:**
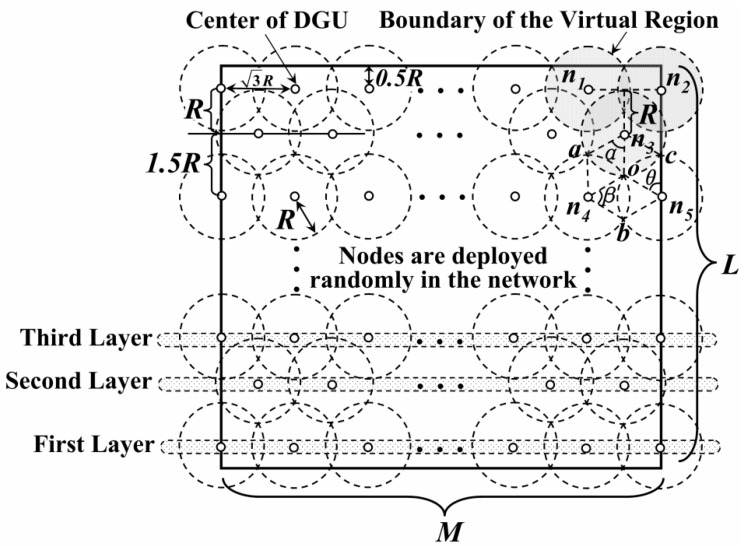
Data gathering unit in the network.

To establish the trajectories of the mobile Sinks and to reduce the amount of redundant data sensed by adjacent nodes, the network is divided into several virtual disks with radius *R.* Each of them is called “Data Gathering Unit” (DGU). Centers of disks (white dots) are distributed on the transverse or longitudinal line, as shown in Figure 1.

According to [1,2,3], data (especially the multimedia data) gathered by nodes closed to each other often show some similarity in the same period. Thus, to decrease the amount of the redundant data, the definition of the “Data Gathering Area” (DGA) is proposed as follows.

As shown in Figure 1, the vertical distance from *n_3_* to the line segment *n_1_n_2_* is just *R*. Thus, the region consisting of DGU *n_1_*, *n_2_* and *n_3_* is defined as a DGA, just as the gray area in Figure 1.

**Figure 2:**
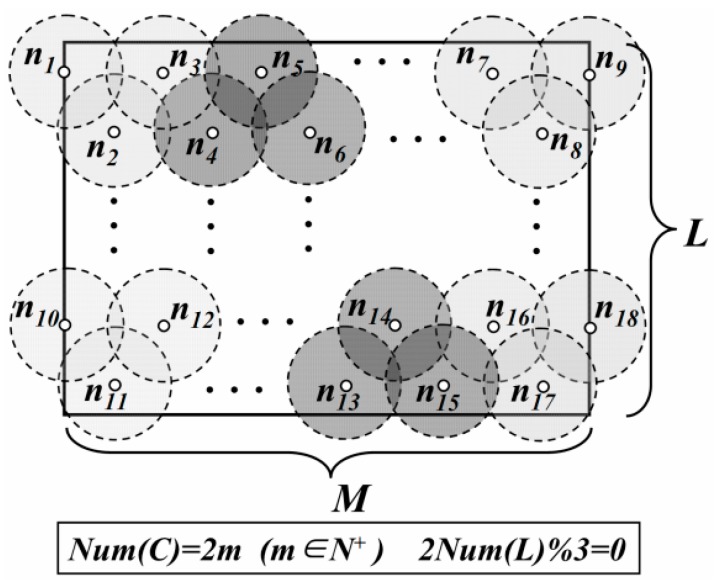
Distribution of DGAs (*Case 1*).

On the other hand, to reduce the probability of collecting redundant data, it is necessary to minimize area of the overlapping region between these DGAs. Thus, in Figure 2, DGU *n_3_*, *n_4_* and *n_5_* need to meet the requirement of the minimum coverage model and *O* is the point where these DGUs intersect. The distance between the centers of these adjacent DGUs is$\sqrt{3}R$. 

In LDGM, the virtual DGUs are uniformly distributed in the network. For the centers of some DGUs, if their vertical coordinates are the same, these DGUs are regarded as in the same layer. Centers with the minimum vertical coordinate value are just located at the first layer. The number of DGUs in the first layer is defined as *Num*(*L*) and *Num*(*C*) is the number of the layers. Then, total number of DGAs (defined as *Num*(*R*)) is

*Case 1*: *Num*(*C*) is even and 2 × *Num*(*L*)%3 = 0 (Figure 2).

(1) $$Num\left(R\right)=\frac{2Num\left(L\right)}{3}\times \frac{Num\left(C\right)}{2}$$

**Figure 3:**
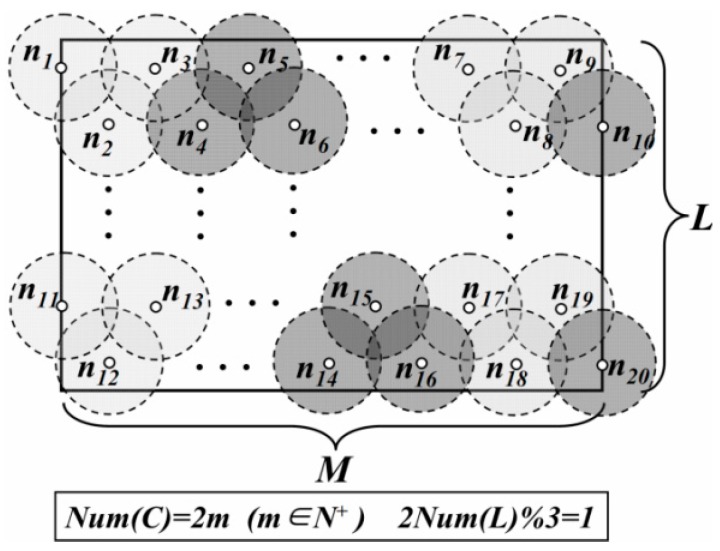
Distribution of DGAs (*Case 2*).

*Case 2*: *Num*(*C*) is even and 2 × *Num*(*L*)%3 = 1 (Figure 3).

(2) $$Num\left(R\right)=\left(\frac{2Num\left(L\right)-1}{3}+1\right)\times \frac{Num\left(C\right)}{2}$$

**Figure 4:**
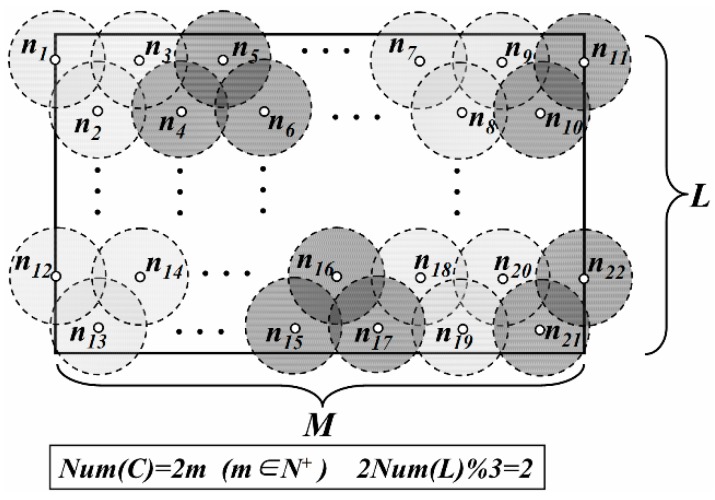
Distribution of DGAs (*Case 3*).

*Case 3*: *Num*(*C*) is even and 2 × *Num*(*L*)%3 = 2 (Figure 4).

(3) $$Num\left(R\right)=\left(\frac{2Num\left(L\right)-2}{3}+1\right)\times \frac{Num\left(C\right)}{2}$$

**Figure 5:**
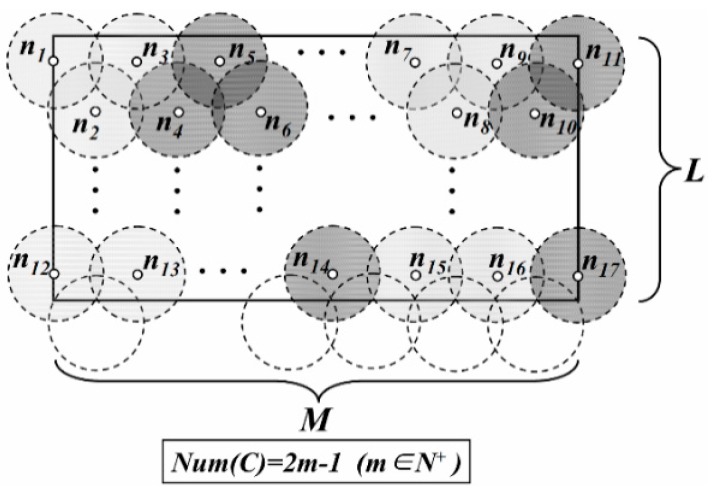
Distribution of DGAs (*Case 4*).

*Case 4*: *Num*(*C*) is odd (Figure 5).

(4) $$Num\left(R\right)=\{\begin{array}{ll} \frac{2Num\left(L\right)}{3}\times P & \sigma =0\\ \left(\frac{2Num\left(L\right)-1}{3}+1\right)\times P & \sigma =1\\ \left(\frac{2Num\left(L\right)-2}{3}+1\right)\times P & \sigma =2 \end{array}\;P=\frac{Num\left(C\right)+1}{2}\;\mathrm{and}\;\sigma =2\times Num\left(L\right)\%3$$

## 4. Data Gathering with the Help of Multi-Sink

### 4.1. Leader Selection Strategy in DGA

In LDGM, it is assumed that one or more “lead nodes” exist in each DGA which receive data from other nodes in the same DGA and upload them to the mobile Sinks. During the network lifetime, the mobile Sinks only need to communicate with these leaders, enhancing the efficiency of data gathering.

**Figure 6:**
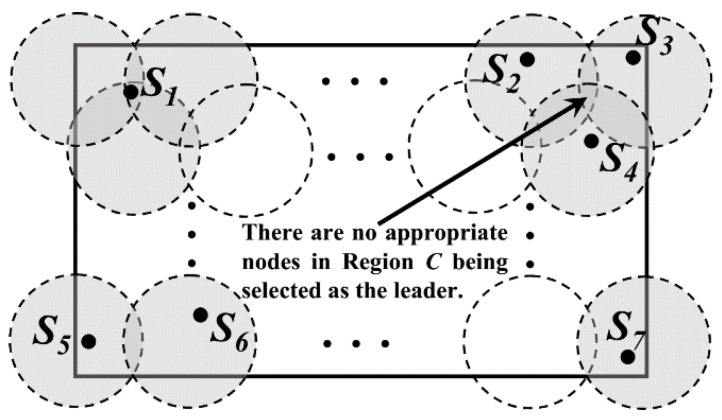
Leader nodes selection in each DGA.

For the DGA consisting of three DGUs, the node located at the triple coverage region (named as “region *C*” for short) could be selected as the leader. Other nodes in the same DGA transmit data to the leader hop by hop. To prolong the network lifetime, node with the maximum residual energy *E_r_* in region *C* is selected as the leader, as *S_1_* shown in Figure 6. The value of *E_r_* must be greater than the threshold, *E_th_*. When the Sinks are far away from the DGA, all of the nodes carry out their sensing tasks and upload data to their leaders.

However, according to the definition of DGA, the area of region *C* is relatively small. So there may be no nodes deployed in this region. In this case, the node with the maximum value of *W* in each DGU will be selected as the leader, as node *S_2_*, *S_3_* and *S_4_* shown in Figure 6. The value of *W* could be calculated as follows: In Formula (5), *d_io_* is the Euclidean distance between node *i* and center of region *C*. *α* and *β* are the adjustable parameters and *α + β = 1*.

(5) $$W=\alpha E_{r}+\beta \frac{1}{d_{io}{}^{2}}$$

Moreover, for the DGA consisting of less than three DGUs, the leaders will be selected directly in each of its DGUs according to Formula (5), as *S_5_*, *S_6_* and *S_7_* shown in Figure 6. If there are just only two DGUs in a DGA, *d_io_* is just the Euclidean distance between node *i* and the midpoint *O* of the line connecting the centers of the two DGUs.

### 4.2. Redundancy Reduction on Data Gathering

As mentioned before, the leader may not be selected from region *C* if the density of nodes in the network is too low. On the contrary, high density of nodes will also cause high redundancy on data collection. Therefore, a type of active node selection strategy and a sensing radius adjustment method are described in this section.

There are two types of sleep scheduling strategies in WSNs, that is centralized and distributed method, respectively [19]. The number of active nodes is relatively small with the help of the centralized sleep scheduling method which could effectively reduce network redundancy. While, time overhead of the centralized method is higher than the distributed one. So, it is not suitable for large-scale sensor networks with high real-time requirement.

**Figure 7:**
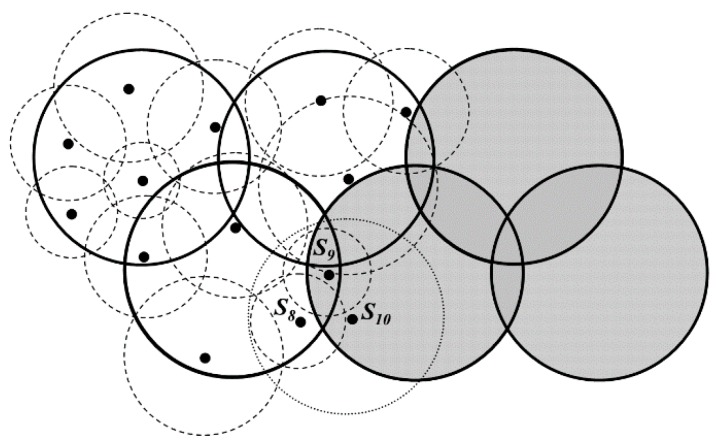
Distributed sleep scheduling strategy in each DGA.

To improve time efficiency in LDGM, the distributed sleep scheduling strategy is executed in each DGA. For example, there are two DGAs in Figure 7 (the white and the gray areas) and the active nodes (black dots) as well as their sensing regions in the white DGA are also shown in this figure. *S_10_* is another active node in the adjacent DGA (the gray one). It is obvious that, both *S_8_* and *S_9_* are active nodes although the sensing areas of them have been completely covered by the sensing area of *S_10_*. Therefore, the effect of the distributed sleep scheduling strategy on redundancy reduction may not be the best. Thus, a sensing radius adjustment strategy is proposed as follows.

#### 4.2.1. Active Node Selection in Each DGA

It is known that, energy consumption on sensing for node *S_i_* (defined as *e_S_*(*S_i_*)) per unit time is related to the length of its sensing radius *R_S_*(*S_i_*) [42]. That is,

(6) $$e_{s}(S_{i})=\lambda_{1}R_{s}{\left(S_{i}\right)}^{\mu }$$

*λ_1_* and *μ* are the adjustable parameters while the residual energy of *S_i_* is defined as *E_r_*(*S_i_*). To prolong network lifetime, we have,

(7) $$e_{s}(S_{i})\times T=\lambda_{2}E_{r}(S_{i})$$

*T* is the expected value of network lifetime and *λ_2_* is the adjustable parameter whose value is between 0 and 1. Thus, with the decrease of the residual energy, length of sensing radius of *S_i_* should also be reduced to conserve its energy. Thus, according to Formulas (6) and (7) we get,

(8) $$R_{s}(S_{i})={\left(\frac{\lambda_{2}E_{r}(S_{i})}{\lambda_{1}T}\right)}^{\frac{1}{\mu }}$$

**Figure 8:**
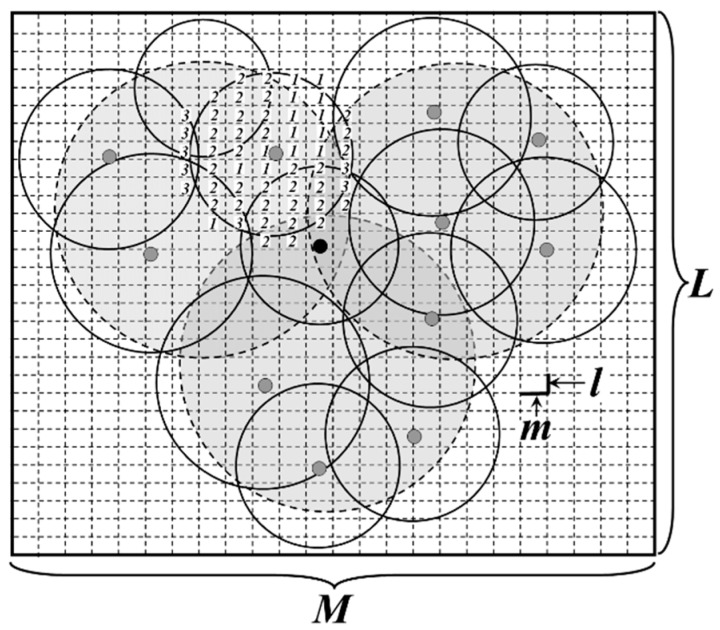
Overlapping sensing area judgment based on grids.

For the convenience of calculation, the network is divided into a number of virtual grids, whose length and width are *m* and *l* respectively, as shown in Figure 8. It is assumed that *m* and *l* could be divisible by *M* and *L*. *g_j_* is defined as the sensing area coverage degree of grid *j*, whose initial value is 0.

At first, all the leaders in each DGA are selected as the active nodes and the length of their sensing radiuses could be calculated by Formula (8). Thus, the value of each *g_j_* now is shown in Figure 8.

Then, other nodes in the same DGA execute the following judgment one by one according to the value of their residual energy *E_r_*(*S_i_*) (from high to low). For each grid *j* that in the area of the circle whose center and radius are *S_i_* and *R_S_*(*S_i_*), if

(9) (xj±m2−x(Si))2+(yj±l2−y(Si))2≤RS(Si)2

Then, *g_j_ = g_j_ +* 1. *x_j_* and *y_j_* are the horizontal and vertical coordinates of the center of grid *j*, while (*x(S_i_)*, *y(S_i_)*) is the coordinate of *S_i_*. As for all the grids in circle *S_i_*, if the value of *g_j_* are all greater than 1, it is considered that the sensing area of *S_i_* has been completely covered by other active nodes. Thus, *S_i_* goes into sleeping mode and in its sensing area, *g_j_ = g_j_ −* 1. Otherwise, *S_i_* becomes the active node.

#### 4.2.2. Sensing Radius Adjustment

It is known that the coverage redundancy of the network could decrease with the help of the active node selection method in Section 4.2.1. However, there are still some active nodes at the boundary of each DGA that could go into sleeping mode, as *S_8_* and *S_9_* shown in Figure 7. Besides, the length of sensing radius *R_S_*(*S_i_*) calculated by Formula (8) may not be optimal. The redundant region may still exist between the adjacent sensing areas. Therefore, sensing radius of the active nodes should be further adjusted. 

**Figure 9:**
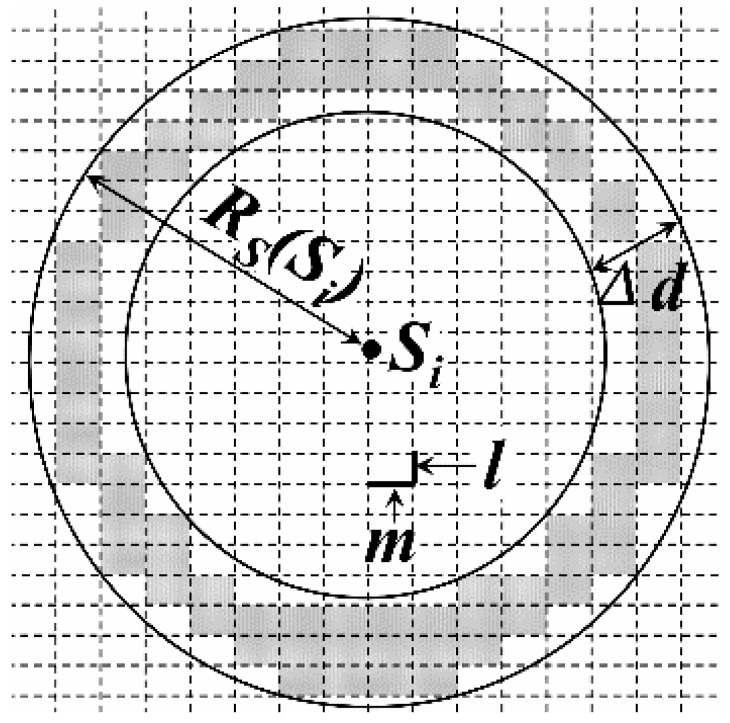
Sensing radius adjustment.

For the grid whose coordinate meets the condition of Formulas (9) and (10), it is known that, it is not in the circle *S_i_* whose radius is *R_S_*(*S_i_*) *−* Δ*d*, as the gray girds shown in Figure 9. In this case, *g_j_ = g_j_ −* 1.

(10) (xj±m2−x(Si))2+(yj±l2−y(Si))2>(RS(Si)−Δd)2

In Formula (10), Δ*d* is a constant and it should meet

(11) $$\Delta d>\sqrt{l^{2}+m^{2}}$$

If there is no grid satisfying *g_j_ = 0* in the annular region whose outer and inner radiuses are *R_S_*(*S_i_*) and *R_S_*(*S_i_*) *−* Δ*d* respectively, we set *R_S_*(*S_i_*) *= R_S_*(*S_i_*) *−* Δ*d*. This judgement process repeats until one of the two following conditions exist:

If there is at least one grid satisfying *g_i_ = 0*, the current value of *R_S_*(*S_i_*) will be set as the sensing radius. Then, we have *g_j_ = g_j_ +* 1 for all the grids in the annular region.

If the value of *R_S_*(*S_i_*) is 0, it is known that the sensing area of *S_i_* is completely covered by that of other active nodes (such as *S_8_* and *S_9_* shown in Figure 7). Then, *S_i_* will go into sleeping mode.

Nodes whose sensing areas are at the boundary of the DGAs adjust the length of their sensing radiuses firstly according to the above steps. For ease of description, it is defined that node which located at the sensing region of *S_i_* and in the same DGA with it is the neighbor of *S_i_*. Thus, the number of neighbors (defined as *Nei*(*S_i_*)) of the node located at the boundary of DGA is often smaller than that of the node inside the DGA. Then we have,

(12) $$\Delta \rho \left(S_{i}\right)=n/M\times L-Nei\left(S_{i}\right)/\pi R_{S}{\left(S_{i}\right)}^{2}$$

If and only if Δ*ρ*(*S_i_*) is greater than a threshold Δ*ρ*, the sensing area of *S_i_* exceeds the boundary of that DGA. In this case, *S_i_* is called the “boundary node”, just as *S_8_*, *S_9_*, *S_10_* shown in Figure 7. The boundary nodes adjust the length of their sensing radiuses one by one according to the value of Δ*ρ*(*S_i_*) (from high to low). It is obvious that the boundary node whose sensing areas are completely covered by other active nodes (including the nodes in other DGAs) goes into sleeping mode after this adjustment. Therefore, the redundancy of data gathered from the same DGA will be decreased.

Subsequently, in each DGA the “non-boundary nodes” follow the same process. Moreover, to save energy as much as possible and to achieve balance of energy consumption, the node with the lower residual energy takes priority to do the sensing radius adjustment.

### 4.3. Energy Efficient Path for Data Transmission in DGA

**Figure 10:**
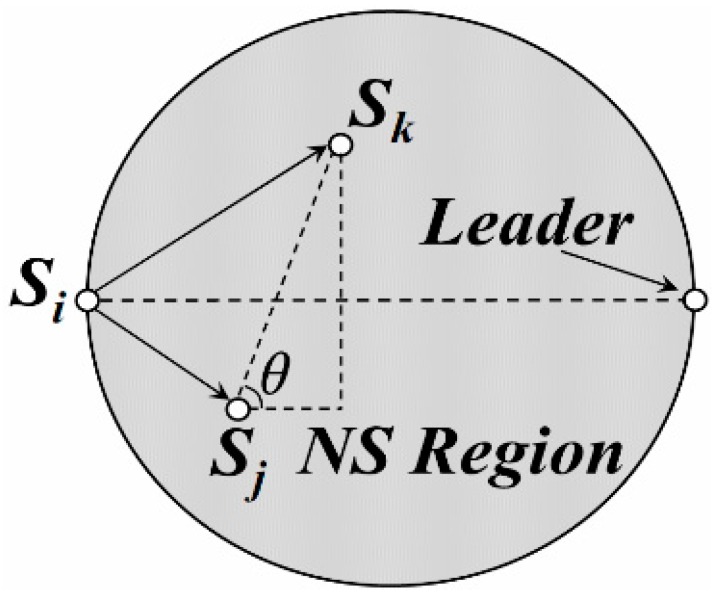
Next-hop node selection in DGA.

Compared with the leader selection and active nodes selection strategy mentioned above, finding the optimized paths for data uploading in each DGA is more important for balancing energy consumption and prolonging network lifetime. As shown in Figure 10, the circular region (length of its diameter is just the straight line distance between *S_i_* and the leader) is regarded as “Region for Next-hop Node Selection” (“*NS Region*” for short). Therefore, if *S_i_* meets Formula (13), it is obvious that there is no active node in “*NS Region*” and *S_i_* transmits its data to the leader by one hop. In Formula (13), *d*(*S_i_,leader*) is the diameter length of the “*NS Region*” and and *ρ_A_* is just the density of active nodes in this region.

(13) $$d\left(S_{i},leader\right)<\sqrt{4/\pi \rho_{A}}$$

Otherwise, when an active node *S_j_* in the “*NS Region*” of *S_i_*, it is regarded as the candidate next-hop node for *S_i_* if and only if it meets all the following requirements.

*Requirement 1*: Residual energy of *S_j_* should be higher than the average value of active nodes in this “*NS Region*”, just as Formula (14) shows. This ensures that *S_j_* has enough energy to transmit the data to the next receiver. *E_r_*(*S_j_*) and *E_r_*(*S_k_*) in Formula (14) are defined as the residual energy of *S_j_* and any active node (e.g., *S_k_*) in this “*NS Region*” respectively. *Num*(*NS*) is the number of active nodes in this region and *λ_4_* is an adjustable parameter whose value is between 0.5 to 1.

(14) $$E_{r}\left(S_{j}\right)\geq \frac{\lambda_{4}}{Num\left(NS\right)}\sum_{k=1}^{Num\left(NS\right)}E_{r}\left(S_{k}\right)$$

*Requirement 2*: Hop distance between *S_j_* and *S_k_* should be shorter than the length of the diameter, while both *S_j_* and *S_k_* are neighbors of *S_i_* in its “*NS Region*”. Thus,

(15) $$d\left(S_{j},S_{k}\right)\leq \lambda_{5}d\left(S_{i},leader\right)$$

*λ_5_* is an adjustable parameter whose value is also between 0.5 to 1.

*Requirement 3*: The “step distance” form *S_j_* to *S_k_* should be longer than 0. As shown in Figure 10, it is just the projection of segment *S_j_S_k_* and its length is defined as,

(16) $$\vec{d'\left(S_{j},S_{k}\right)}=d\left(S_{j},S_{k}\right)\cos\theta$$

As for all the candidate next-hop nodes (e.g., *S_j_*, *S_k_*, …), the weight of each path *S_j_S_k_* is defined as,

(17) $$W\left(S_{j},S_{k}\right)=2E_{elec}+\varepsilon_{fs}d^{2}\left(S_{j},S_{k}\right)$$

**Figure 11:**
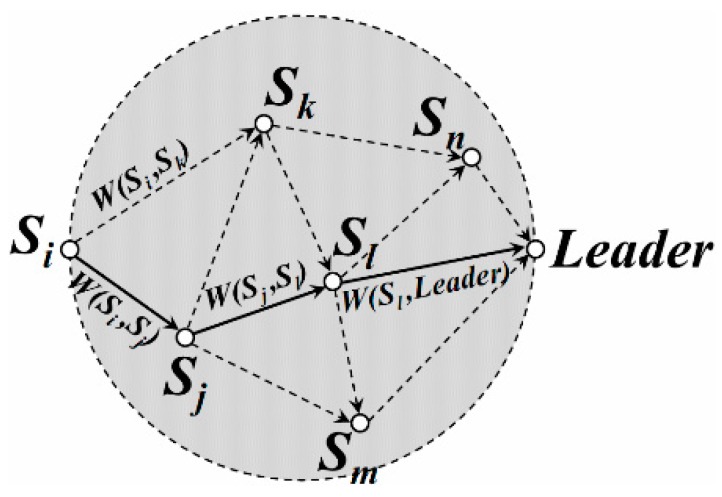
Weighted directed graph for data uploading.

*W*(*S_j_,S_k_*) is just the energy consumption on transmitting one bit of data from *S_j_* to *S_k_*. Path *S_j_S_k_* is more likely to be one of the uploading paths from *S_i_* to the leader when this weight is relatively small. Thus, a weighted directed graph could be constructed in the “*NS Region*”, as shown in Figure 11. And the Single-Source Shortest Path form *S_i_* to the leader could also be found (as the black solid line in Figure 11) with the help of the Dijkstra algorithm. This is the energy efficient data uploading path for *S_i_*.

### 4.4. Moving Path for Multi-Sink

**Figure 12:**
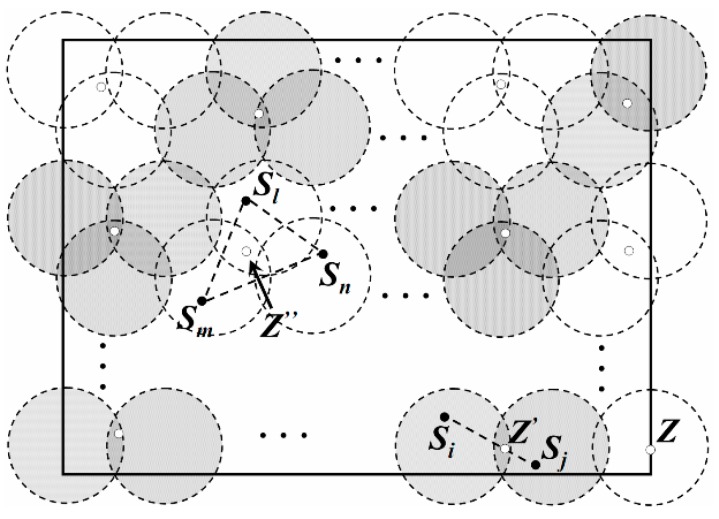
Location of the traverse point.

In LDGM, there is one Traverse Point (TP) in each DGA (the white dots in Figure 12) and the mobile Sinks stay at each TP for a period of time to receive data from the leader (the black dots in Figure 12). Then, they move to the next TP at a fixed speed *v*. So, the whole trajectory for one Sink in a data collection cycle is composed of *Num(R) −* 1 segments.

As for a DGA, when the leader is located at region *C*, the traverse point is just the center of this region. Therefore, distance between the leader and the mobile Sink is relatively shorter that decreases energy consumption on communication.

When the leaders are located at each DGU, the location of the traverse point could be calculated as follows:

*Case 1:*  If the DGA is composed of a single DGU, the traverse point is just the center of this DGU. As point *Z* shows in Figure 12*.*
*Case 2:*  If there are only two DGUs in the DGA, it is known that two leaders exist in this DGA (just *S_i_* and *S_j_* in Figure 12). It is assumed that, *Z’* is the TP of this DGA while *k_i_* and *k_j_* are the data being uploaded from the leaders to the mobile Sink. So, total energy consumption for *S_i_* and *S_j_* on data uploading could be described as follows.
  (18) $$E_{T}\left(S_{i}\right)+E_{T}\left(S_{j}\right)=\left(k_{i}+k_{j}\right)E_{elec}+k_{i}\varepsilon_{fs}d^{2}\left(S_{i},Z'\right)+k_{j}\varepsilon_{fs}d^{2}\left(S_{j},Z'\right)$$
  To get the minimum value of *E_T_*(*S_i_*) *+ E_T_*(*S_j_)*, the optimal location of *Z’* could also be calculated with the help of the partial derivative, as shown in (19)*, X*(*S_i_*)*,Y*(*S_i_*) and *X*(*S_j_*)*,Y*(*S_j_*)*,* are the coordinates of *S_i_* and *S_j_* respectively.
  (19) $$\begin{array}{l} X\left(Z'\right)=\frac{k_{i}X\left(S_{i}\right)+k_{j}X\left(S_{j}\right)}{k_{i}+k_{j}}\\ Y\left(Z'\right)=\frac{k_{i}Y\left(S_{i}\right)+k_{j}Y\left(S_{j}\right)}{k_{i}+k_{j}} \end{array}$$
*Case 3:*  If there are three DGUs in the DGA (e.g., *S_l_*, *S_m_* and *S_n_* are the leaders), the coordinates of its TP (e.g., *Z’’*) could be calculated out by Formula (20).
  (20) $$\begin{array}{l} X\left(Z''\right)=\frac{k_{l}X\left(S_{l}\right)+k_{m}X\left(S_{m}\right)+k_{n}X\left(S_{n}\right)}{k_{l}+k_{m}+k_{n}}\\ Y\left(Z''\right)=\frac{k_{l}Y\left(S_{l}\right)+k_{m}Y\left(S_{m}\right)+k_{n}Y\left(S_{n}\right)}{k_{l}+k_{m}+k_{n}} \end{array}$$

**Figure 13:**
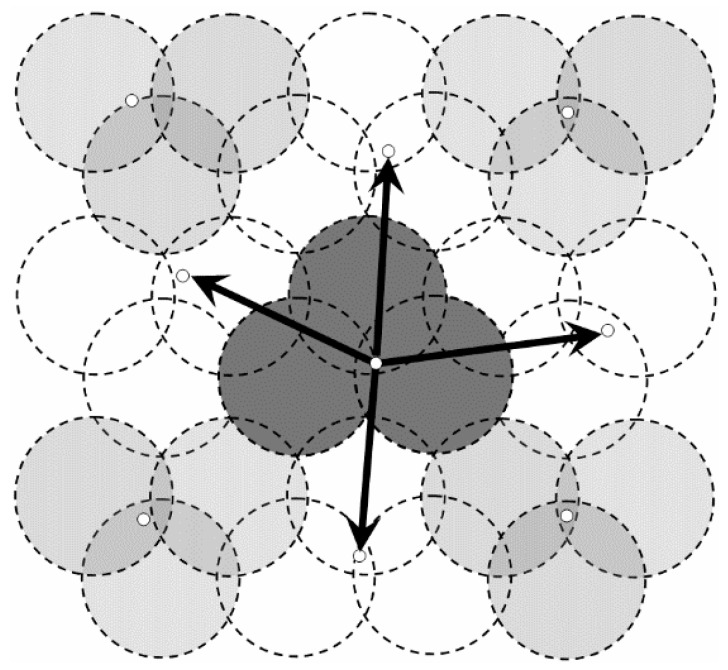
The next available TPs for selection.

For a DGA (e.g., *DGA_i_*), if another DGA (e.g., *DGA_j_*) whose region is partly shared with the region of *DGA_i_*, these two DGAs are regarded as neighbors. So, each DGA has at most four neighbors. As shown in Figure 13, the DGA and its neighbors are colored with black and white, respectively.

For any mobile Sink, if the distance between its two consecutive TPs is too long, its energy consumption on moving will higher. In this case, the length of the trajectory will also increase and may cause “buffer overflow problems”. As shown in Figure 12, the mobile Sink is now at *Z’’* and its next TP is *Z*. Thus, in the next period of time, the leaders in the neighbor DGAs of the DGA where *Z’’* is located may have no chance to upload data to this mobile Sink. Therefore, some constraints are described as follows.

*Constraint 1*: There are *k* mobile Sinks in the network.
*Constraint 2*: For a Sink at *DGA_i_*, the next TP of it could only be selected from the TPs in the neighbors *DGA*.
*Constraint 3*: During one data gathering period *T*, each Sink should move to each TP once and only once.

As for a mobile Sink (e.g., *Sink*(*i*)) locating at *TP*(*m*), the weight that *Sink*(*i*) moves to the next TP (e.g., *TP*(*n*)) at current time *t* is defined as follows.

(21) $$P_{TP\left(m\right)\_TP\left(n\right)}\left(t\right)=\left(t-t_{p}-t_{0}\right)\sum_{i=1}^{Num\left(DGA_{n}\right)}\pi R_{S}{}^{2}\left(S_{i}\right)u_{i}$$

In Formula (21), *t_p_* is the time when *TP*(*n*) was visited by another Sink last time. *Num*(*DGA_n_*) is regarded as the number of active nodes in the DGA where *TP*(*n*) locates at. *πR_S_^2^*(*S_i_*)*u_i_* is just the data gathering rate of each active node *S_i_* whose sensing radius is *R_S_*(*S_i_*).

When *Sink*(*i*) stays at *TP*(*m*) for a period time slot *t_0_*, other TPs in the neighbor DGAs calculate the value of their weight*.* The TP with the maximum value of *P_TP(m)_TP(j)_* and has not yet being visited by *Sink*(*i*) is selected as the next TP of *Sink*(*i*).

If there is no such traversal points, *Sink*(*i*) moves back to the previous traverse point *TP*(*k*) and chooses one of the unvisited TPs with the maximum weight value from the neighbor DGAs as its next TP.

Thus, the hop distance of the mobile Sink will not be too long and the probability that TPs in the neighboring DGAs being visited by *Sink*(*i*) will also be increased. Moreover, the amount of data waiting for uploading in one DGA is another important factor in determining which TP *Sink*(*i*) will visit next, just as Formula (21) shows. Therefore, the possibility of buffer overflow on leaders could be effectively decreased.

It is also known that the longest period of time that the leader could cache data in DGA_n_ could be calculated as follows.

(22) $$T_{th}\left(n\right)=Cache/\sum_{i=1}^{Num\left(DGA_{n}\right)}\pi R_{S}{}^{2}\left(S_{i}\right)u_{i}$$

Without loss of generality, the value of the parameter *Cache* is set to 4 KB (just the buffer size of MicaZ). Thus, for the network with *k* mobile Sinks, the period *T* for one round of data collection is defined as follows.

(23) $$T=MAX\left(Num\left(R\right)t_{0}+\frac{1}{v_{\eta }}\sum_{j=1}^{Num\left(R\right)-1}l_{j}|\eta =1,2,...k\right)$$

Parameter *t_0_* is defined as the time slot that Sink stays at each TP and *v_η_* is the moving speed of Sink(*η*). While *l_j_* is the straight line distance between two adjacent TPs on the trajectory of one Sink. So the value of *T* in Formula (23) is just the longest time that the Sink spends on visiting all the TPs.

For one DGA, if the time intervals between any two successive arrivals of different Sinks could be approximately equal, the efficiency of LDGM could be further improved and the burden on the leader could also be reduced. In this case, the time interval *T_o_’* is,

(24) To'=1kMAX(Num(R)t0+1vη∑j=1Num(R)−1lj|η=1,2,...,k)

To avoid buffer overflow, the value of *T_o_’* should meet,

(25) To'≤MAX(Tth(n)|n=1,2,...,Num(R))

So, with the help of Formulas (22)–(24), we get,

(26) $$k\geq \frac{MAX\left(Num\left(R\right)t_{0}+\frac{1}{v_{\eta }}\sum_{j=1}^{Num\left(R\right)-1}l_{j}|\eta =1,2,...,k\right)}{Cache/MAX\left(\sum_{i=1}^{Num\left(DGA_{n}\right)}\pi R_{S}{}^{2}\left(S_{i}\right)u_{i}|n=1,2,...,Num\left(R\right)\right)}$$

However, in LDGM, trajectories of the mobile Sinks are not fixed and their moving paths could also be affected by the value of *P_TP(m)_TP(n)_*(*t*). Thus, *T_o_’* is just the optimal value of time interval. In the worst case, no Sinks visit the DGA during the time of *T-kt_0_*, so the longest time interval *T_m_’* is,

(27) Tm'=MAX(Num(R)t0+1vη∑j=1Num(R)−1lj|η=1,2,...,k)−kt0

In this case, if and only if *T_m_’* satisfies the following conditions, the cache overflow does not occur.

(28) Tm'≤MAX(Tth(n)|n=1,2,...,Num(R))

Accordingly, from Formulas (22), (27) and (28), we could also get,

(29) $$\begin{array}{cc} k\geq & \frac{1}{t_{0}}MAX\left(Num\left(R\right)t_{0}+\frac{1}{v_{\eta }}\sum_{j=1}^{Num\left(R\right)-1}l_{j}|\eta =1,2,...,k\right)\\ & -\frac{Cache}{t_{0}MAX\left(\sum_{i=1}^{Num\left(DGA_{n}\right)}\pi R_{S}{}^{2}\left(S_{i}\right)u_{i}|n=1,2,...,Num\left(R\right)\right)} \end{array}$$

With the help of Formulas (26) and (29), the optimal number of mobile Sinks deployed in the network could be calculated out.

## 5. Simulation Results

To evaluate the performance of LDGM on balancing energy consumption, prolonging network lifetime as well as reducing coverage redundancy and transmission delay, a series of simulations are carried out with the help of VC++6.0 and Matlab 8.5. We also compared LDGM with some typical cluster based data gathering methods with mobile Sinks, e.g., MST, MWST and so on. Values of the parameters in these experiments are shown in Table 1.

### 5.1. Network Coverage and Redundancy

**Figure 14:**
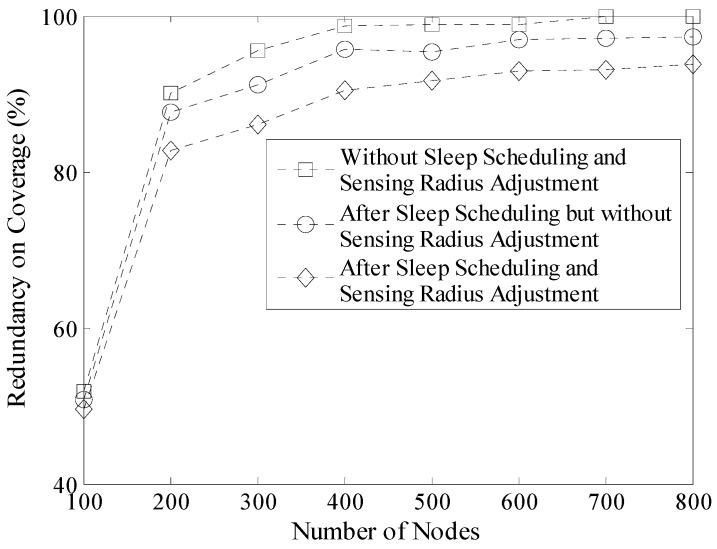
Redundancy on coverage (100 m × 100 m).

To evaluate the performance of LDGM on reducing redundancy on coverage, experiments are carried out in the following cases. Network size is 100 m × 100 m and the results of these experiments are shown in Figure 14.

*Case 1*: Nodes are deployed randomly in the network without doing any optimization.
*Case 2*: Nodes in the network carry out sleep scheduling strategy after being deployed.
*Case 3*: Nodes in the network execute both of the sleep scheduling and the sensing radius adjustment algorithms.

Network coverage rate under different number of nodes is shown in Table 2. As mentioned in Section 4, LDGM does not change the network coverage rate. So when the number of nodes is fixed, the coverage rates in the above three cases are the same. Without loss of generality, the “virtual grids” described in Section 4.2 is used to calculate the network redundancy on coverage (defined as *η*). *Q_i_* = 1 when *g_i_ >* 1, otherwise *Q_i_ =* 0.

(30) $$\eta =\left(m\times l\times \sum_{i=1}^{\left(M/m\right)\times \left(N/n\right)}Q_{i}\right)/\left(M\times L\right)$$

In Figure 14, it is known that with the rise of the number of nodes, redundancy on coverage also increases. Moreover, the increasing rate is faster when the number of nodes increases from 100 to 400. At the same time, the coverage rate has also enhanced from 79.8% to 99.9%. This is the main reason why the redundancy increases sharply. However, after doing sleep scheduling as well as sensing radius adjustment, the redundancy on coverage decreases a lot (in some cases, more than 10%).

**Figure 15:**
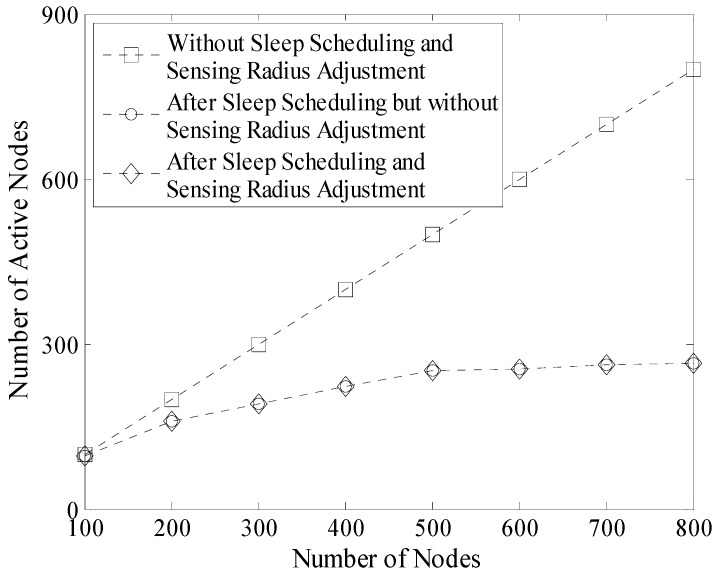
Number of active nodes in LDGM (100 m × 100 m).

Figure 15 shows the number of active nodes before and after sleep scheduling. According to the description in Section 4, it is known that the node whose sensing area is completely covered by other nodes will be in sleep mode without reducing the network coverage rate. Therefore, as shown in Figure 15, the number of active nodes is significantly decreased after sleep scheduling. Moreover, this number is nearly stable (no more than 260 in this experiment) when the number of nodes increases.

It is necessary to mention that by carrying out a series of experiments, we found that the number of active nodes was not significantly reduced. This is because only the nodes near the boundary of the DGA would go into the sleeping mode during the sensing radius adjustment process. Thus in Figure 15, the two broken-lines with circular mark and diamond mark almost coincide.

**Figure 16:**
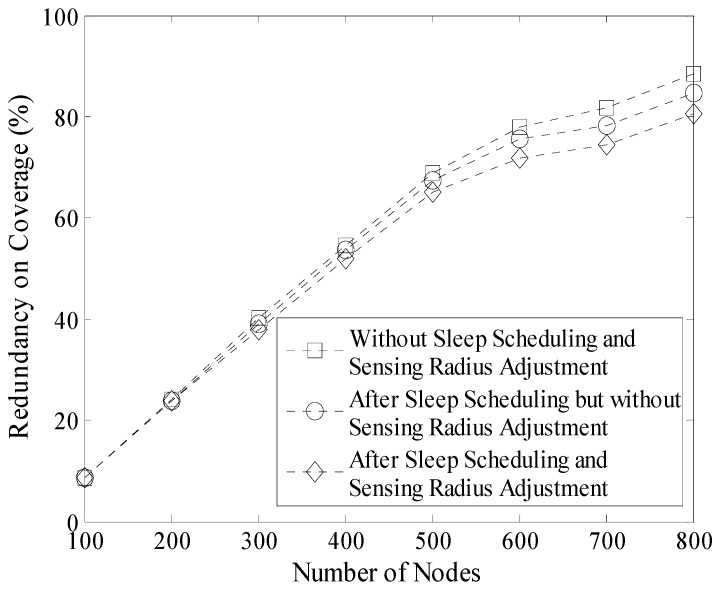
Redundancy on coverage (200 m × 200 m).

**Figure 17:**
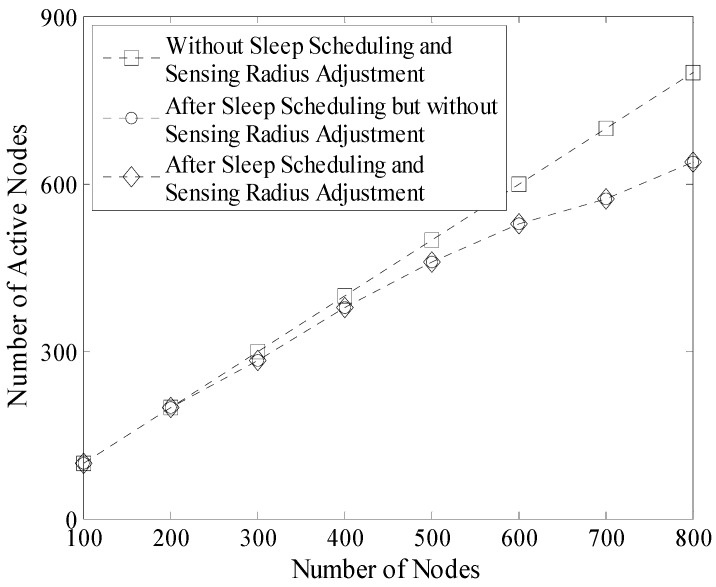
Number of active nodes in LDGM (200 m × 200 m).

Experiment results in the 200 m × 200 m network are shown in Figure 16, Figure 17 and Table 3 respectively. Compared with the result shown in Figure 15, it is known that the decrease of the redundancy on coverage in Figure 16 is not obvious. Because in this case density of nodes is lower and the initial redundancy is also lower than that in the network whose size is 100 m × 100 m. In addition, coverage rate of the network does not decrease in LDGM. So, there are still many active nodes in this network (see Figure 17).

**Figure 18:**
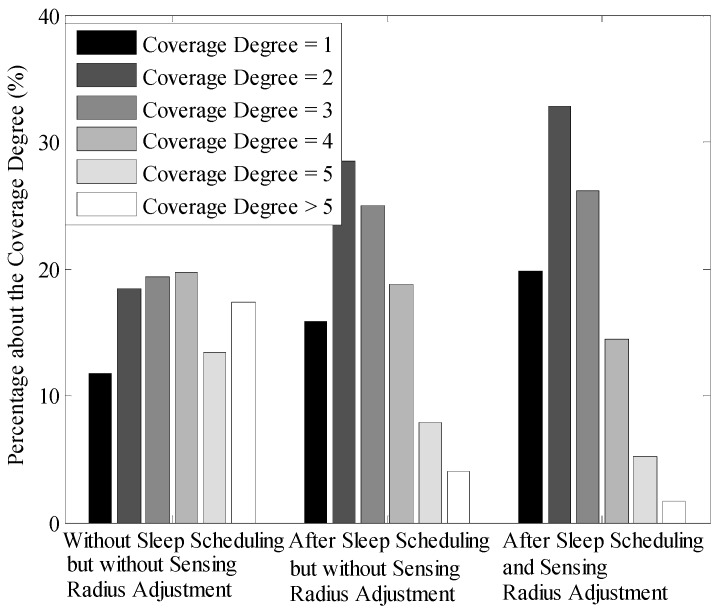
Percentage about the coverage degree (100 m × 100 m, 200 nodes).

As shown in Table 2, the network coverage rate is up to 97% when 200 nodes are randomly deployed in the network whose size is 100 m × 100 m. Accordingly, we further analyze the coverage degree of the network in LDGM by using the same values of these parameters and the result is shown in Figure 18. It is known that the coverage degree over most of the redundant area (over 60%) is more than three when the nodes are randomly deployed, while this percentage is obviously reduced after carrying out the sleep scheduling algorithm. Moreover, the coverage degree of more than 70% of the redundant area in the network is no more than three after sensing radius adjustment.

Lengths of the sensing radius after adjustment are shown in Table 4. The initial sensing radius lengths are set to 5 m, 6 m, 7 m and 8 m respectively in these experiments and each value of *λ_2_* could be calculated out by Formula (7). It is obvious that no matter what the value of the initial sensing radius is, there are always more than 50% nodes that shorten their sensing radius after the adjustment process in LDGM. Among them, more than 70% nodes shorten their sensing radius by 1–3 m effectively reducing the amount of redundant sensing data as well as energy consumption on sensing.

### 5.2. Average Residual Energy of Nodes

**Figure 19:**
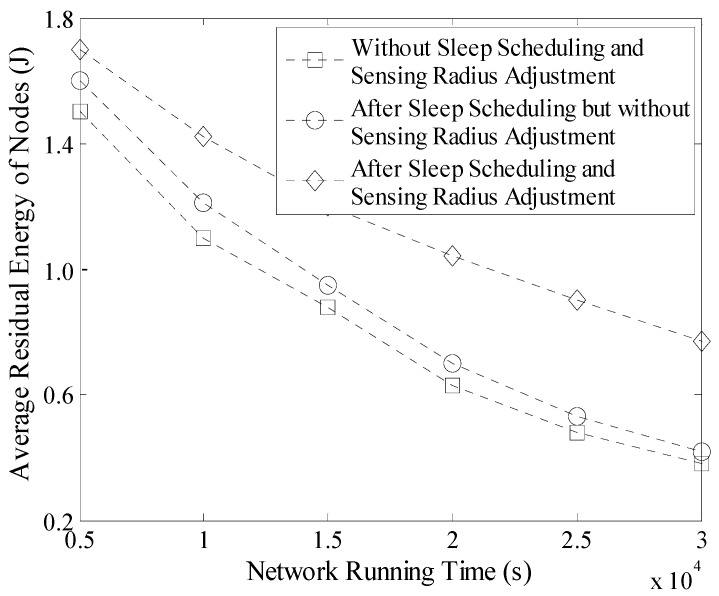
Average residual energy of nodes (100 m × 100 m, 500 nodes).

The Average Residual Energy (ARE) of 500 nodes in a 100 m × 100 m network is shown in Figure 19 and there are four mobile Sinks in this network for data gathering. It is shown that the residual energy decrease quickly when no optimization algorithms are carried out. At the time of 2 × 10^4^ seconds this value is already below the threshold, 0.7 J. Because in this case, all nodes are active and they collect data with their initial sensing radius which may lead to high energy consumption. Moreover, the leaders should upload a considerable number of redundant data to the mobile Sink that also shortens the network lifetime.

While after sleep scheduling, the number of active nodes significantly decreases, so the value of ARE increases. Moreover, after sensing radius adjustment, network life in LDGM could be further prolonged. For example, at the time of 3 × 10^4^ s, the value of ARE is closed to 0.8 J which ensures that most of the nodes could still work for a long time.

### 5.3. Time Delay in Data Gathering

As mentioned above, in LDGM each Sink is required to move to each traverse point once and only once during one round of data gathering period. Thus for any *TP_j_*, the maximum time interval (defined as *TP_j_*(Δ*t_max_*)) between two consecutive visits is,

(31) $$TP_{j}\left(\Delta t_{\max}\right)=MAX\left(t_{i+1}^{j}-t_{i}^{j}|i=1,2,...,k-1\right)$$

*t_i_^j^* refers to the time when *Sink(i)* moves to *TP_j_* while *k* is the number of Sinks. Therefore, the maximum time delay on data gathering could be defined as,

(32) $$\Delta T_{\max}=MAX\left(TP_{j}\left(\Delta t_{\max}\right)|j=1,2,...,Num\left(R\right)\right)$$

Similarly, average time delay on data collection is defined as,

(33) $$\Delta T_{avg}=\sum_{j=1}^{Num\left(R\right)}TP_{j}\left(\Delta t_{\max}\right)/Num\left(R\right)$$

**Figure 20:**
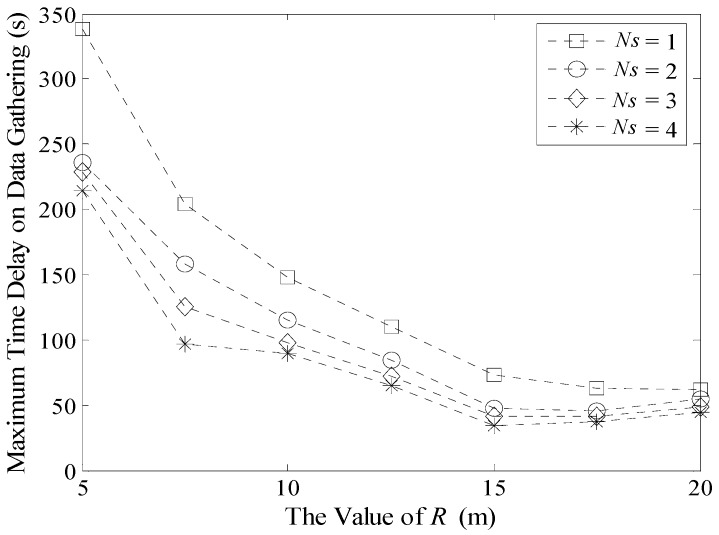
Maximum time delay on data gathering (100 m × 100 m, 200 nodes).

**Figure 21:**
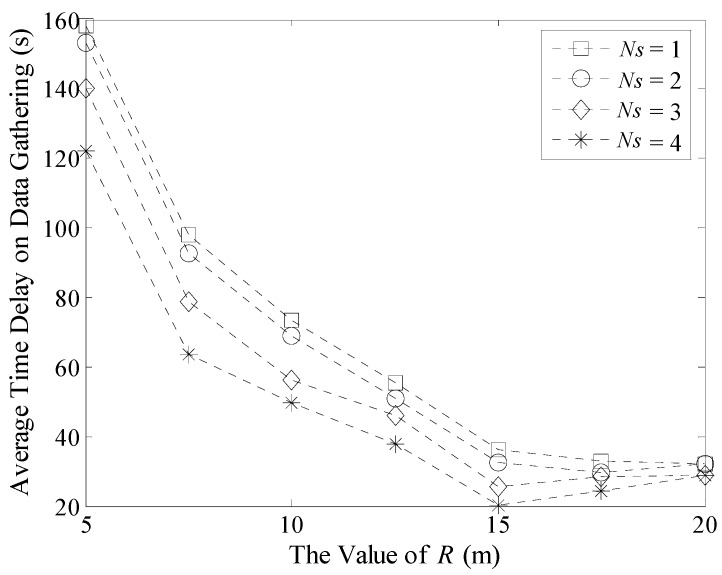
Average time delay on data gathering (100 m × 100 m, 200 nodes).

Experiment results of Δ*T_max_* and Δ*T_avg_* with different *R* and *N_s_* in a 100 m × 100 m network are shown in Figure 20 and Figure 21. When *N_s_* is fixed, the value of Δ*T_max_* decreases with the increase of *R*. This is because when *R* is larger, there will be fewer DGAs in the network and the number of TP will decrease. Thus, the total time spent by delaying at these traverse points also decreases time spent during data gathering.

On the other hand, when the value of *R* is fixed, time delay in a multi-Sink based network is much smaller than the network that has only one Sink. However, decrease in time delay is not obvious when the number of Sinks increases from three to four. Because when there are more Sinks moving in the network, the possibility that different Sinks visit the same TP at the same time or during a short time interval increases. That is, the value of *TP_j_*(Δ*t_max_*) increases according to Formula (31). As a result, Δ*T_max_* is stable.

When the value of *R* is larger, there will be less traverse points in the network. Thus, the effect on reducing time delay is not very obvious no matter how many Sinks are moving in the network. As shown in Figure 20, when *R* is 20 m, the maximum time delays on data gathering with different Sinks are nearly the same. Therefore, the time efficiency of LDGM will be better when the network size is large and *R* is small.

Figure 21 shows the average time delay on data gathering. In the majority of cases, the effect on multi-Sink based network is better than that with only one Sink. Moreover, when the value of *R* is small, this advantage is more obvious. While if *R* is larger, Δ*T_avg_* increases slightly, as shown in Figure 20.

**Figure 22:**
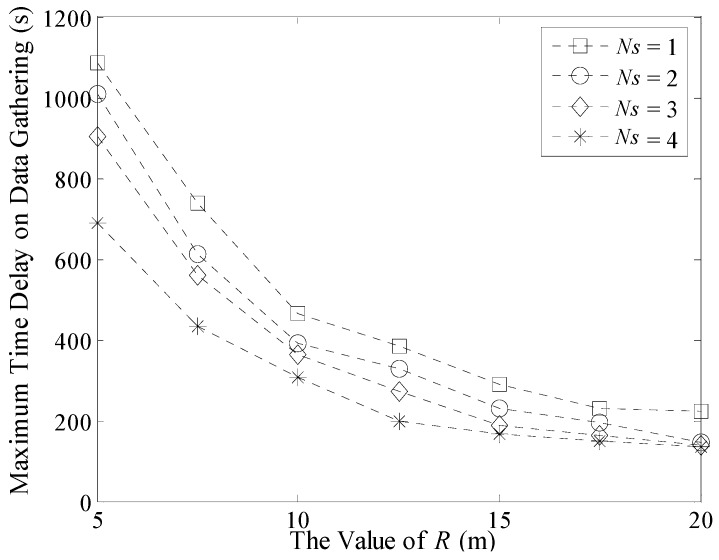
Maximum time delay on data gathering (200 m × 200 m, 500 nodes).

**Figure 23:**
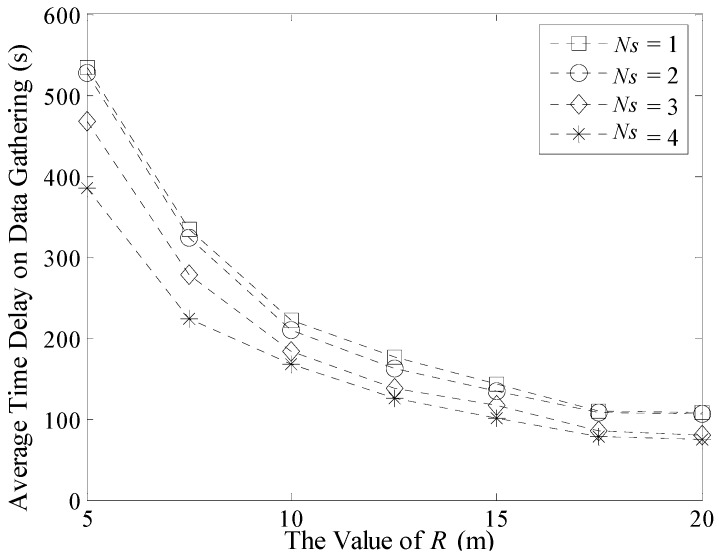
Average time delay on data gathering (200 m × 200 m, 500 nodes).

In addition, experiments about the maximum and average time delay in a 200 m × 200 m network are shown in Figure 22 and Figure 23. Similar to Figure 20, the maximum time delay decreases with the increase of *R* when the number of Sinks is fixed. However in Figure 22, when *R* increases from 15 to 20, this value still decreases which is different from the result in Figure 20. This is because the number of TPs in Figure 22 is obviously more than that in the 100 m × 100 m network. In this case, the possibility that two different Sinks visiting the same TP during a short time interval is not large. So the value of Δ*T_max_* could still decrease.

Similarly, the average time delay in a multi-Sink based network is obviously shorter than that in the single Sink based one, as shown in Figure 23. The value of it decreases slowly with the increase of *R*.

**Figure 24:**
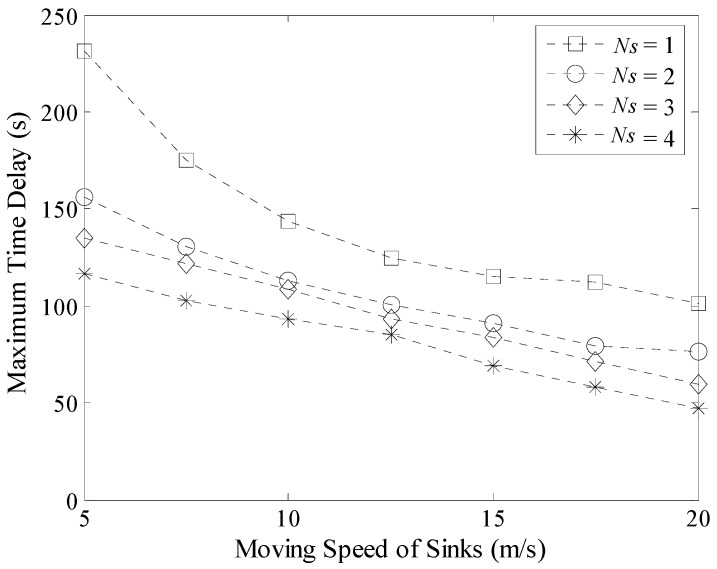
Maximum time delay with different moving speeds of Sinks.

**Figure 25:**
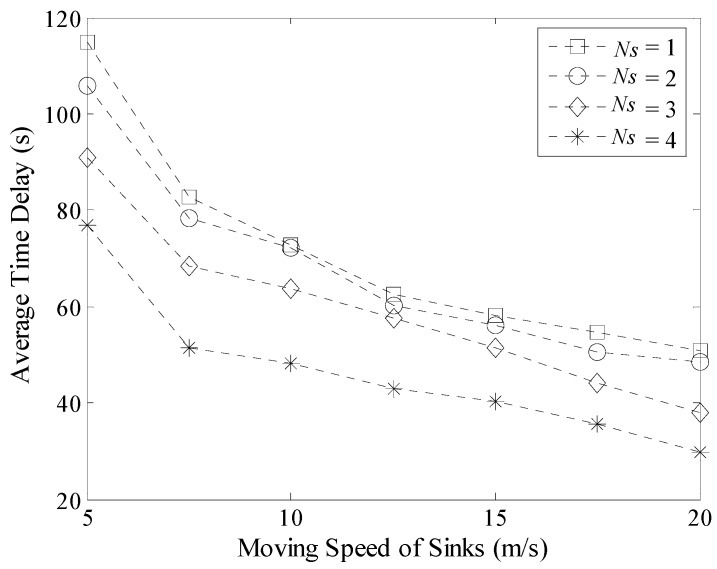
Average time delay with different moving speeds of Sinks.

Experiment results about the maximum and average time delay with different moving speeds of Sinks are shown in Figure 24 and Figure 25 respectively. When the value of *v* increases, the delay decreases in LDGM. However, this decreasing rate becomes slower and slower when the speed of Sink further increases. This is because the length of time the mobile Sink is staying at each TP is a constant value in LDGM. In this case, the increase of speed could only shorten the time spent on moving. In addition, no matter how fast the moving speed is, time efficiency of the multi-Sink based network is always higher than that of single Sink based network.

### 5.4. Efficiency of Data Gathering

In LDGM, the leader carries a big load its energy consumption is much higher than any other node. However, there are no differences in size of cache as well as the ability to transmit data between these two types of nodes. So how to speedily upload data to the mobile Sink and how to avoid a buffer overflow on leaders are also important problems requiring resolution.

**Figure 26:**
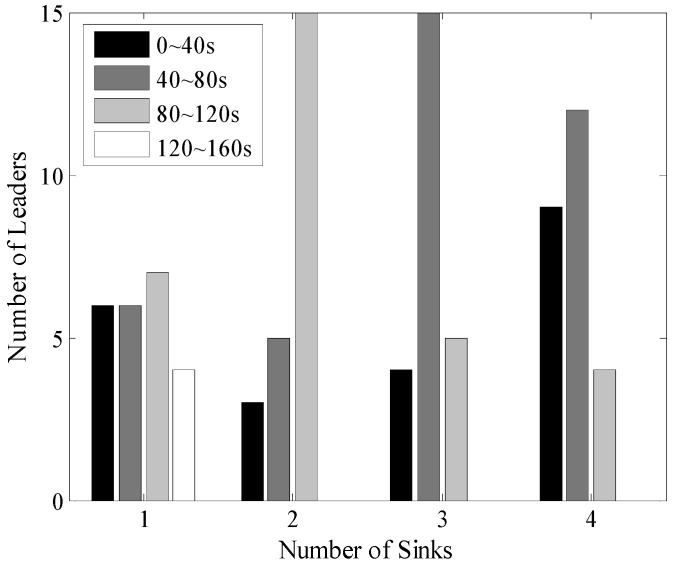
Time interval of two consecutive times of data upload (100 m × 100 m, 200 nodes).

**Figure 27:**
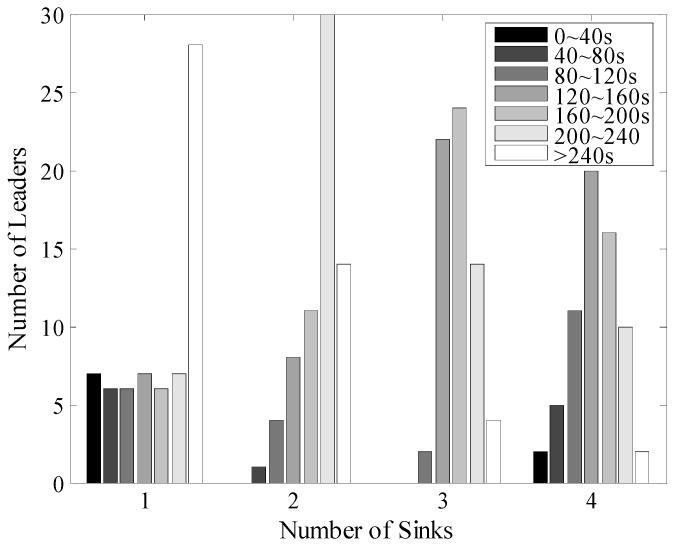
Time interval of two consecutive times of data upload (200 m × 200 m, 500 nodes).

Firstly, we analyze the time interval of the two consecutive data uploading process of each leader with the size of the networks set to 100 m × 100 m and 200 m × 200 m, as shown in Figure 26 and Figure 27. In the single Sink based network, each TP could only be visited once during one round of data gathering time. So, the distribution of these time intervals is nearly uniform. It should be pointed out that the different moving paths of Sinks could also influence the length of time intervals but after many experiments, it was found that the impact is limited. While with the increase of *N_s_*, the time interval between two consecutive times of data uploading is obviously shorter for example, in the 100 m × 100 m network with four mobile Sinks for more than 80% of the leaders, the time intervals are shorter than 120 s. Thus, LDGM could greatly increase the frequency of data uploading and therefore reduce the probability of buffer overflow.

**Figure 28:**
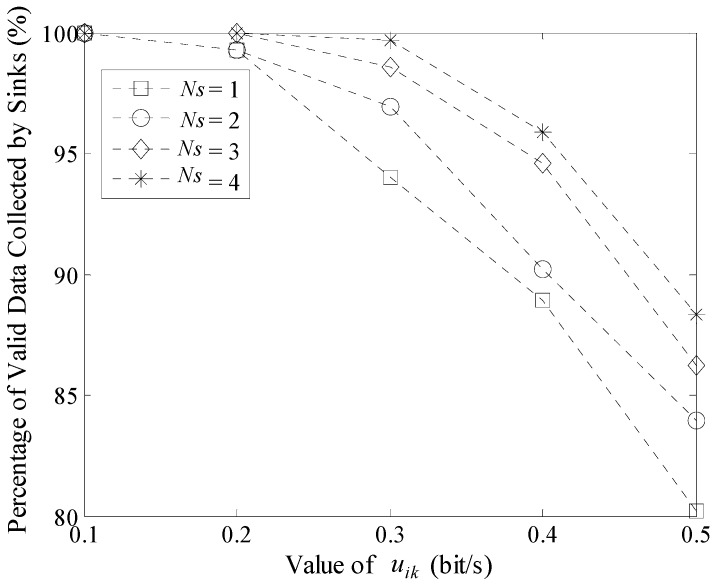
Percentage of valid data collected by Sinks (100 m × 100 m, 200 nodes).

Moreover, Percentage of Valid Data collected by Sinks (PVD) is another important indicator to show the efficiency of data gathering. PVD in LDGM is defined as the ratio between the amount of data received by all the mobile Sinks and the total amount of data collected by all active nodes in a round of data gathering time. Experimental result is shown in Figure 28. With the increase of *u_ik_*, the value of PVD decreases. In LDGM, the cache size of each node is set to 4KB so it will overflow when the sensing rate is too high. On the other hand, PVD will be higher when *N_s_* is larger, e.g., when *N_s_* = 4, even if *u_ik_* is up to 0.5 bit/(m^2^·s), the value of PVD is still nearly 90%.

### 5.5. Costs on Data Gathering

**Figure 29:**
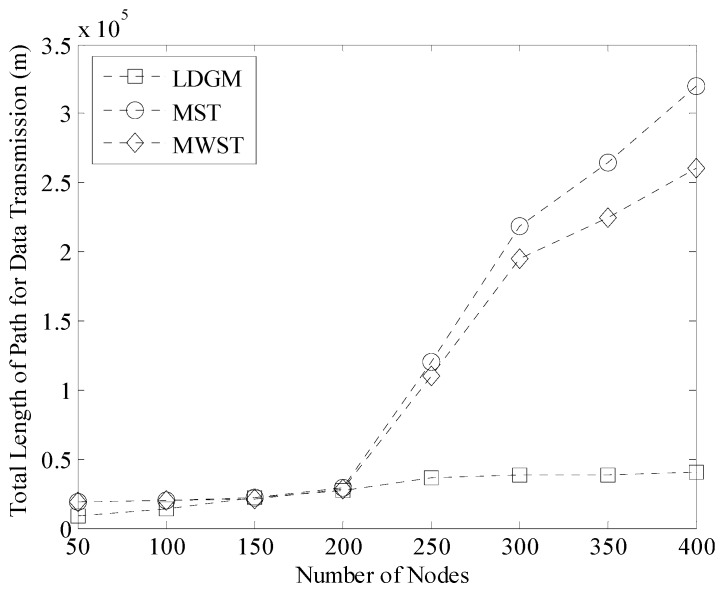
Total length of path for data transmission (100 m × 100 m, 200 nodes).

To further verify the performance of LDGM on total length of data transmission path as well as network energy consumption, we compared it to MST (a shortest path based data gathering method) and MWST (a type of backbone based data collection strategy with the help of randomly moving Sink). Figure 29 shows the total length of Path for Data Transmission (PDT) during one round of data gathering time. It is known that the length of PDT in MST is the longest. Because each node in the network needs to do a continuous sensing task and upload data directly to Sink. Thus, MST performs well if and only if there are not too many nodes in the network. When the density of nodes is larger, the length of PDT obviously increases.

**Figure 30:**
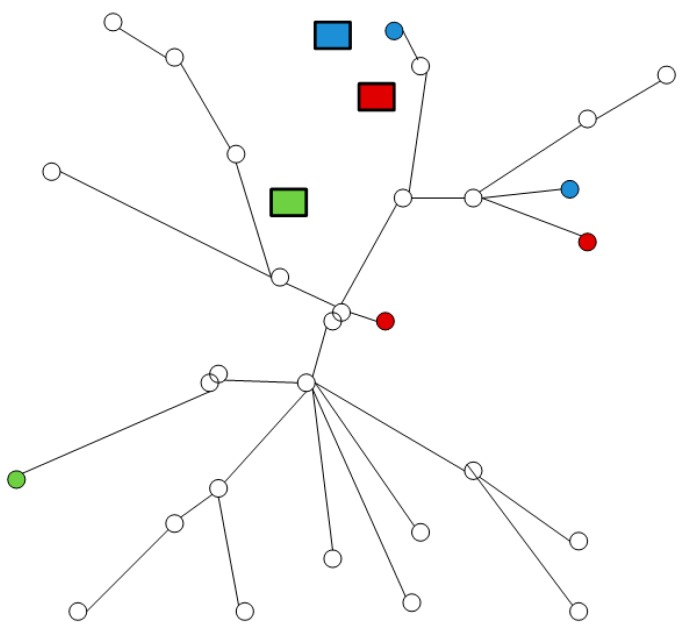
Data collection path of MWST.

MWST is another efficient data gathering method by finding out the shortest uploading path. With the help of the mobile Sink, not all nodes need to transmit data to the receiver directly. Thus, the length of PDT in MWST is shorter than that in MST. However, the mobile Sink moves randomly along the backbone path. Other nodes need to upload data to Sink by single-hop or multi-hop manner. As shown in Figure 30, rectangles with different colors are the mobile Sinks. Each Sink needs to visit the nodes that have the same color as itself. So its length is still much longer than that of LDGM.

While in our algorithm, each active node only needs to upload data to the leader closest to it, so the length of PDT in LDGM is the shortest. Moreover, trajectories of mobile Sinks in LDGM are controllable. These Sinks visit each TP and receive data from the leader that shorten the distance of single-hop transmission. In addition, the advantage of LDGM on the length of PDT is more obvious when the number of nodes is over 200, as shown in Figure 29. In this case, the number of active nodes is still stable with the help of the sleep scheduling and sensing radius adjustment processes. Thus, the total length of paths for data transmission in LDGM does not significantly increase.

**Figure 31:**
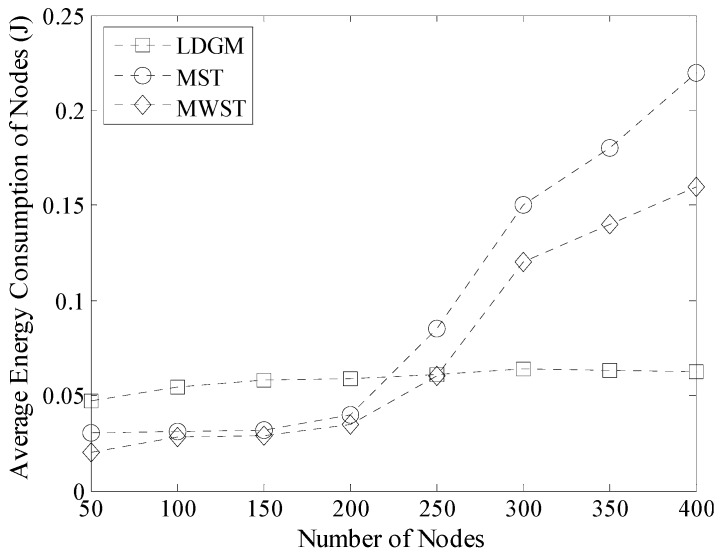
Average energy consumption of nodes (100 m × 100 m, 200 nodes).

Average energy consumption of nodes is shown in Figure 31. It is known that, the advantage on energy saving in LDGM is not very obvious when there are a small amount of nodes deployed in the network because the total lengths of PDT in MST and MWST are short when there are not too many nodes in the network (see Figure 30). Moreover, in this case, the hop distances between nodes and Sink are also short in both of these two algorithms. Thus, according to the data transmission model proposed by Heinzelman [19], it is known that, energy consumption on communication in MST and MWST is low.

In LDGM, there are fewer nodes in each DGA when the number of nodes is less than 200. In this case, most of the nodes prefer to upload data to the leader by using a single-hop way which enhances their energy consumption. However, with the help of the sensing radius adjustment strategy, energy consumption on sensing is greatly decreased for most active nodes. So, total energy consumption of LDGM is still of the same level as that of MST and MWST.

With the increase of the density of nodes, the advantage of LDGM on energy saving is more and more obvious and the average energy consumption of nodes is also stable because in this case, the number of active nodes increases slowly by carrying out the sleep scheduling strategy. Moreover, the optimal path for data transmission in each DGA could also be found out, which restrains the increase of energy consumption in LDGM.

**Figure 32:**
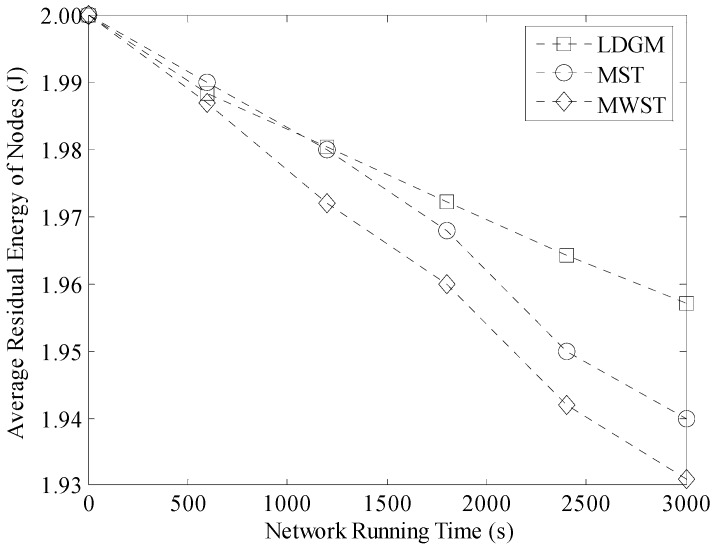
Average residual energy of nodes (100 m × 100 m, 200 nodes).

Average residual energy of nodes in the three types of data gathering algorithms is shown in Figure 32. Similar to Figure 31, when the density of nodes in the network is large, the transmission paths in MST and MWST are longer. Thus, residual energy of nodes is lower. With the help of one mobile Sink, the residual energy of some nodes decreases slowly in MWST. But this Sink moves randomly in the network. It may move to a hotspot area that could increase the energy consumption on nodes in this region. However, in LDGM, each active node only needs to upload data to the leader in the same DGA. So, total length of the transmission paths is not too long. In addition, the leader selection and the sleep scheduling strategies further balance energy consumption in our algorithm. Thus, average residual energy of nodes in LDGM is higher than MST and MWST.

## 6. Conclusions

To achieve energy balance in sensor networks, a type of multi-Sink based data gathering method is proposed in this paper. By selecting the optimal leader node in each DGA, the energy efficient data uploading paths could be established and trajectories of Sinks are also optimized improving time efficiency in data collection. Moreover, with the help of the sleep scheduling and the sensing radius adjustment strategies, redundancy in network coverage and energy consumption in sensing and communication could also be effectively reduced.

Currently in the big data era, time efficiency is topic under close review. The time delay in data gathering has been reduced with the help of multiple Sinks in LDGA, but different Sinks may move to the same traversal point simultaneously, extending the time delay in data uploading in some DGA. It should be further improved in our future work.

## Figures and Tables

**Table 1:** Parameter values in simulation.

Parameter	Symbol	Value	Unit
Network Size	*M × L*	100 × 100~200 × 200	m^2^
Radius of DGU	*R*	5~20	m
Number of Nodes	*N*	100~800	
Number of Mobile Sinks	*Ns*	1–4	
Weight Coefficient	*α*	0.2	
Weight Coefficient	*β*	0.8	
Adjustable Parameter	*λ_1_*	2 × 10^−6^	J/(m^2^·s)
Sensing Rate	*u_ik_*	0.05~0.5	bit/(m^2^·s)
Data Collection Rate of the Mobile Sink	*v’*	100	Kbps
Moving Speed of the Mobile Sink	*v*	5–20	m/s
Initial Energy of Nodes	*E_0_*	2.0	J
Threshold of the Residual Energy	*E_th_*	0.7	J
Buffer Size of One Node	*Cache*	4	KB
Energy Consumption of Sending and Receiving Circuit	*E_elec_*	50	nJ·b^−1^
Energy Consumption of Amplifier in Free-Space Model	*μ_fs_*	10	pJ·(b/m^2^)^−1^
Energy Consumption of Amplifier in Multi-Path Fading Model	*μ_amp_*	0.0013	pJ·(b/m^4^)^−1^

**Table 2:** Network coverage rate (100 m × 100 m).

Number of Nodes	*N =* 100	*N =* 200	*N =* 300	*N =* 400
Coverage Rate	0.798059	0.970983	0.988334	0.999804
Number of Nodes	*N =* 500	*N =* 600	*N =* 700	*N =* 800
Coverage Rate	0.995491	0.996863	0.999902	1.000000

**Table 3:** Network coverage rate (200 m × 200 m).

Number of Nodes	*N =* 100	*N =* 200	*N =* 300	*N =* 400
Coverage Rate	0.374174	0.5835	0.717433	0.826786
Number of Nodes	*N =* 500	*N =* 600	*N =* 700	*N =* 800
Coverage Rate	0.904532	0.946486	0.955843	0.973194

**Table 4:** Lengths of the sensing radius after adjustment.

Value of *λ_2_*	Number of Active Nodes	*R_s_*(*S_i_*) after Amended	Number of Nodes with Different *R_s_*(*S_i_*)
6.4 × 10^−5^	252 (Initial Value of *R_s_*(*S_i_*) is 8 m)	7 m	155
		6 m	51
		5 m	11
4.9 × 10^−5^	284 (Initial Value of *R_s_*(*S_i_*) is 7 m)	6 m	179
		5 m	28
		4 m	3
3.6 × 10^−5^	317 (Initial Value of *R_s_*(*S_i_*) is 6 m)	5 m	168
		4 m	22
		3 m	2
2.5 × 10^−5^	382 (Initial Value of *R_s_*(*S_i_*) is 5 m)	4 m	182
		3 m	12
		2 m	0
